# Alzheimer’s disease biomarkers in patients with obstructive sleep apnea hypopnea syndrome and effects of surgery: A prospective cohort study

**DOI:** 10.3389/fnagi.2022.959472

**Published:** 2023-01-17

**Authors:** Weili Kong, Yi Zang

**Affiliations:** ^1^Department of Information Management, West China Second University Hospital, Key Laboratory of Birth Defects and Related Diseases of Women and Children, Ministry of Education, Sichuan University, Chengdu, China; ^2^Department of Otolaryngology, Head and Neck Surgery, West China Hospital, Sichuan University, Chengdu, China

**Keywords:** uvulopalatopharyngoplasty, Alzheimer’s disease, biomarker, cognitive function, somnolence

## Abstract

**Background:**

Obstructive sleep apnea hypopnea syndrome (OSAHS) may cause Alzheimer’s disease (AD), t-tau, p-tau, Aβ42, and Aβ40 are important elements in the process of AD, and changes in the levels of these biomarkers may affect the cognitive functioning of patients. Our objective was to investigate whether uvulopalatopharyngoplasty could reduce the plasma levels of AD biomarkers in OSAHS patients and the potential correlations of AD biomarkers with cognitive impairment and sleepiness, and explore the independent influencing factors of cognitive function.

**Methods:**

Alzheimer’s disease biomarkers were measured in the plasma of 35 patients with severe OSAHS requiring surgical treatment and 16 healthy controls without OSAHS. The cognitive function and sleepiness of OSAHS patients was also evaluated. The case group was given uvulopalatopharyngoplasty and followed at the postoperative sixth month, the follow-up cases were 27, and plasma AD biomarker levels, cognitive function, and sleepiness were re-evaluated. The preoperative and postoperative AD biomarker levels OSAHS patients were compared with each other and those of the control group. Linear stepwise regression and lasso regression were used to explore the relationships of AD biomarkers with cognitive impairment and sleepiness.

**Results:**

Significantly higher Aβ40, t-tau, p-tau in plasma were observed preoperatively in OSAHS patients comparing to controls (29.24 ± 32.52 vs. 13.18 ± 10.78, *p* = 0.049; 11.88 ± 7.05 vs. 7.64 ± 4.17, *p* = 0.037; 26.31 ± 14.41 vs. 17.34 ± 9.12, *p* = 0.027). The sixth month of postoperation, the plasma AD biomarkers (Aβ42, Aβ40, t-tau, p-tau) in plasma levels decreased significantly (0.23 ± 0.17 vs. 0.20 ± 0.16, *p* = 0.0001; 29.24 ± 32.52 vs. 23.52 ± 24.46, *p* = 0.0046; 11.88 ± 7.05 vs. 8.88 ± 6.21, *p* = 0.0001;26.31 ± 14.41 vs. 20.43 ± 10.50, *p* = 0.0001). A comparison of MMSE and ESS scores from before to after surgery revealed obvious differences (27.14 ± 1.65 vs. 29.07 ± 1.78, *p* = 0.0001; 11.91 ± 4.84 vs. 5.89 ± 2.83, *p* = 0.0001). Changes in cognitive function and sleepiness scores from before to after uvulopalatopharyngoplasty were significantly correlated with AD biomarkers. Body mass index and t-tau were potential influencing factors cognitive function.

**Conclusion:**

Obstructive sleep apnea hypopnea syndrome can increase plasma AD biomarkers levels. Uvulopalatopharyngoplasty can improve patients’ cognition and sleepiness, and the mechanism may be related to changes in plasma AD biomarkers. Higher AHI and higher t-tau level were identified as independent risk factors for cognitive decline.

## Introduction

Obstructive sleep apnea hypopnea syndrome (OSAHS) can damage many systems in the body and is considered a substantial risk factor for various neurocognitive disorders and cardiovascular diseases (Emamian et al., 2016). Preclinical studies demonstrated that untreated obstructive sleep apnea syndrome may cause Alzheimer’s disease (AD) (Pan et al., 2014; Andrade et al., 2018). Some studies identified OSAHS as a potentially reversible cause of cognitive impairment and suggest that early treatment of OSAHS may decelerate the progress of dementia (Ancoli-Israel et al., 2008; Cooke et al., 2009; Troussière et al., 2014).

The pathological mechanism of AD involves the formation of senile plaque by extracellular deposits of amyloid β protein in nerve tissues. Hyperphosphorylation of tau protein leads to neurofibrillary tangles that form paired helical filaments, causing nerve cell death (Mayeux, 2010). T-tau, p-tau, Aβ42, and Aβ40 are important elements in the process of AD (Zetterberg et al., 2010), and changes in the levels of these biomarkers may affect the cognitive functioning of patients. In a meta-analysis, Kang et al. (2022) proposed that OSAHS was related to changes of AD-related markers, and the severity of the disease affected the development of AD. AD-related biomarkers may be useful for detecting OSAHS and related cognitive impairments (Kang et al., 2022). OSAHS is a risk factor of AD, and the lack of OSAHS following uvulopalatopharyngoplasty (UPPP) may improve AD biomarkers.

Continuous positive airway pressure (CPAP) and UPPP have proven beneficial effects on the cognition and sleep of OSAHS patients. However, little is known about whether UPPP causes changes in the AD biomarker levels of OSAHS patients or whether these changes affect cognitive function and sleep. For this study, our research objectives were to investigate whether plasma AD biomarker levels differ between OSAHS patients and controls, whether UPPP could reduce the plasma AD biomarker levels in these patients and improve cognition and sleep, and whether it is related to changes in AD biomarkers. Furthermore, we explored whether accumulated Aβ42, Aβ40, t-tau, and p-tau in plasma are risk factors for cognitive impairment.

### Patients and methods

**FIGURE 1:**
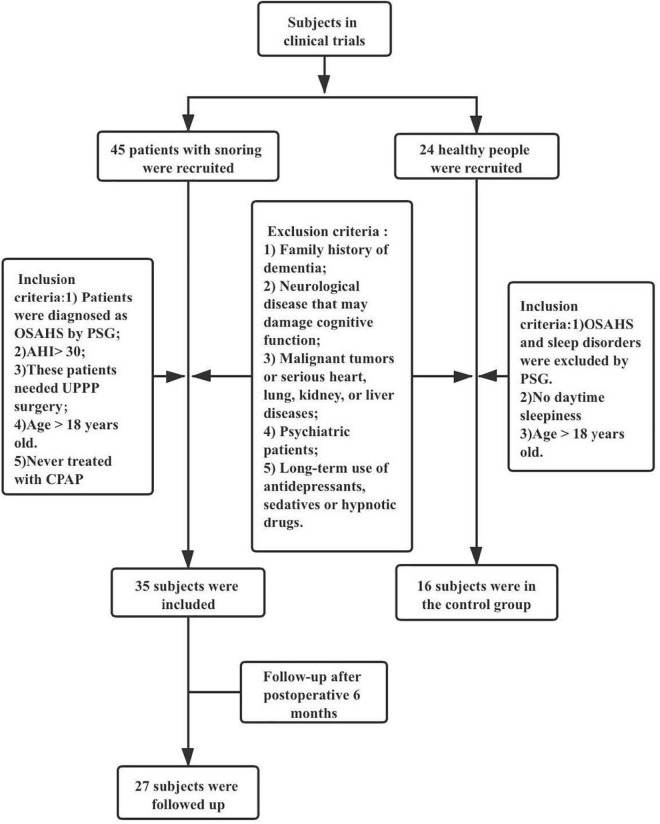
Trial profile.

This prospective study included 35 patients diagnosed with OSAHS at West China Hospital from May to July 2019 (We defined this group as case group). All patients were treated with UPPP, and 27 attended follow-up appointments 6 months postoperatively. Additionally, a control group comprised 16 normal cases without OSAHS (the group found to have normal sleep by PSG and were age- and gender-matched to the preoperative group). Inclusion criteria: (a) patients diagnosed as OSAHS by polysomnography (PSG); (b) OSAHS was classified as severe (AHI>30); (c) After examination (imaging, laryngoscope), it was confirmed that the obstruction site was in the pharyngeal cavity plane and UPPP operation was required; (d) Age over 18; and (e) These patients had not been treated by CPAP. The exclusion criteria: (a) family history of dementia; (b) neurological disease (e.g., Parkinson’s disease) that may damage cognitive function; (c) malignant tumors or serious heart, lung, kidney, or liver diseases; (d) psychiatric patients; and (e) long-term use of antidepressants, sedatives or hypnotic drugs (Figure 1).

All subjects provided written informed consent.

## Data acquisition

### Preoperative basic information

We recorded each patient’s age, gender, height, weight, blood pressure, smoking history, drinking history, medication history, special disease history, surgical history, body mass index (BMI), blood routine, blood biochemistry, coagulation function, arterial blood gas, and lung function tests.

### Subjective evaluation of sleepiness symptoms and global cognitive function

The experimental group completed the Epworth Sleepiness Scale (ESS) and Mini Mental State Examination Scale (MMSE) on the 1 day before surgery and the sixth month after surgery.

### Polysomnography respiratory monitoring

All patients were subjected to PSG using the Alice5 device (Philips Wellcome, USA)^1^, including 6 electroencephalogram electrodes, 2 eye electrodes, 3 mental myoelectric electrodes, 4 lower limb myoelectric electrodes, 3 cardiac electric electrodes, oronasal airflow thermal sensor, nasal pressure tube, thoracoabdominal bandage, snoring monitor, and oxyhemoglobin saturation monitor. PSG monitoring results were interpreted by qualified doctors and technicians. The main indexes including Total sleep time (TST), Oxygen desaturation index (ODI), Apnea hypopnea index (AHI), Rapid eye movement (REM). TST refers to the time from the start of sleep to the end of the last sleep stage minus the time to be awake during sleep. AHI was defined as the average total number of apnea and hypopnea events per hour of sleep. ODI was defined as the number of decreases in mean oxygen saturation per hour of sleep over 3% from baseline. All patients had severe OSAHS (AHI > 30), which was in reference to the guidelines of Vishesh et al. (2017).

### Blood sample collection and preservation

Fasting blood was collected from 06:00 to 07:00 per subject, avoiding circadian rhythm-related changes as much as possible. Blood samples of the experimental group were taken 1 day before surgery and three to 6 months after surgery. For the control group, fasting blood was collected from 06:00 to 07:00 on the next morning after PSG monitor. 2 ml of fresh blood was collected into a sterile anticoagulation tube. The collected blood was centrifuged in a centrifuge at 4°C and 2500 rpm for 10 min. All samples were centrifuged after venous blood sampling, the supernatant was stored in a refrigerator at −80°C.

### AD biomarker testing

Fasting blood samples were collected from the experimental group on the 1 day before surgery and sixth month after surgery. All ELISA kits were from ThermoFisher, Inc.^2^ Plasma Aβ40 ranged from 0 to 100 pg/mL, Aβ42 ranged from 0 to 8000 pg/mL, t-tau ranged from 0 to 1000 pg/mL, and p-tau ranged from 0 to 2000 pg/mL (Figure 1). All ELISA test procedures were in accordance with the manufacturer’s instructions. The kit was taken out of the refrigerator in advance for rewarming at normal temperature, and the plasma was taken out of the −80°C refrigerator and placed on ice. The standard curves of the four proteins were tested before the start of the test sample. All ELISAs were carried out in strict accordance with the manufacturer’s instructions. Each sample was measured at least 3 times according to the standard, and the average value was taken for the statistical analysis.

### Statistical analysis

Descriptive statistics were provided with mean and standard deviation (SD) for continuous variables, and frequency and proportions for categorical variables. We used paired tests to compare differences (before and after operation) in AD biomarker levels between the preoperative group and postoperative group. For the comparison between preoperative group and control group, between postoperative group and control group, Wilcoxon rank sum test was conducted. We used a linear regression analysis to explore correlations between changes in the MMSE and ESS scores and Aβ42, Aβ40, t-tau, and p-tau. Spearman correlation coefficients were used to assess the correlation between the biomarkers and MMSE and ESS. In order to explore the risk factors that may affect cognitive functions, we applied linear stepwise regression model and lasso regression model. For all comparisons, two-sided tests were applied with *P* < 0.05 considered as the statistical significance. All statistical analyses and figures were conducted in R (version 3.5.1) software and Prism 8.0.

## Results

The average age of OSAHS patients was 39.18 ± 9.22 years, and this group had a significantly higher BMI than that of the control group (*p* = 0.003). There were no significant inter-group differences in age, gender, blood pressure, and diabetes prevalence. In contrast, the plasma Aβ 40, t-tau, and p-tau levels were significantly higher in the OSAHS group than in the control group (*p* < 0.05; Table 1).

**TABLE 1:** Characteristics of the study population.

Clinical characteristics	Case group (*n* = 35)	Control group (*n* = 16)	*P*
Age (years, SD)	39.18 ± 9.22	42.43 ± 9.79	0.26
Gender (male,%)	30 (85.8%)	14 (87.5%)	0.84
Education (University degree,%)	4 (11.4%)	2 (12.5%)	0.93
BMI (kg/m2)	25.65 ± 2.78	23.02 ± 2.70	0.003
Hypertension (yes,%)	14 (40%)	6 (37.5%)	0.87
Diabetes (yes,%)	1 (1%)	0 (0%)	1.00
Total sleep time (min, SD)	442.8 ± 75.09	384.2 ± 115.7	0.11
REM (min, SD)	63.28 ± 36.5	102.3	0.04
AHI (times, SD)	63.92 ± 23.65	2.62 ± 1.93	0.0001
LSaO2 (%)	71.91 ± 13.71	87.25 ± 6.93	0.008

**FIGURE 2:**
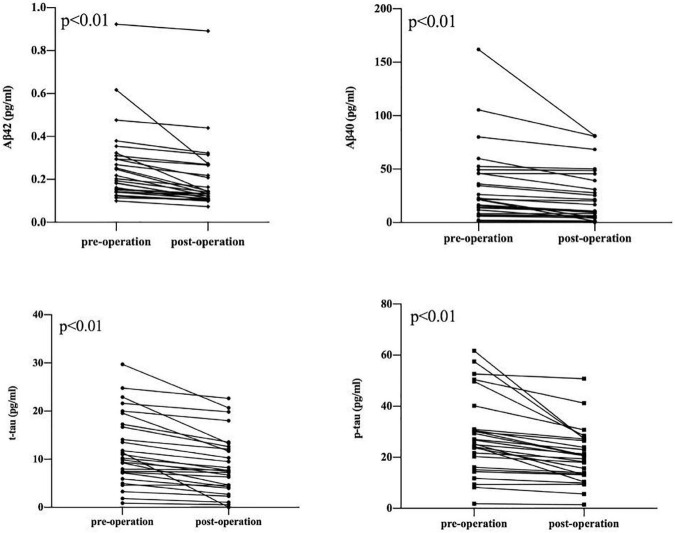
Decrease in Aβ 42, Aβ 40, t-tau, p-tau between pre-operation group and post-operation group. The symbols ● and ▪ represent the value of the patient’s biomarker.

**FIGURE 3:**
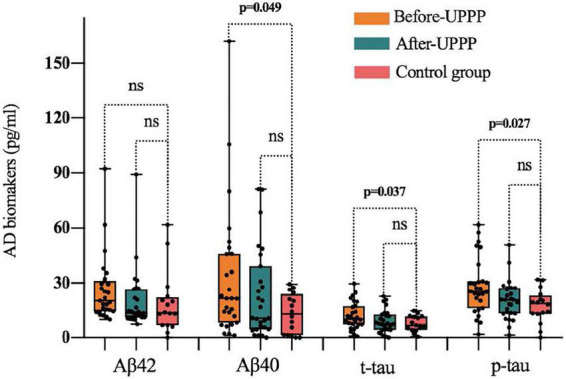
Difference of Aβ 42, Aβ 40, t-tau, p-tau in preoperative, postoperative, and control group; ns is for not significant (*p* > 0.05). The symbol ● represents the value of the patient’s biomarker.

A comparison of changes in AD biomarker levels and MMSE and ESS scores from before to after surgery revealed obvious differences (*p* < 0.05). The postoperative plasma AD biomarkers were significantly lower than the preoperative levels in OSAHS patients (Table 2 and Figure 2). Preoperative AD biomarkers were significantly higher than those in the control group, while the postoperative AD biomarkers decreased to close to the control group (Table 3 and Figure 3).

**TABLE 2:** Differences of Mini Mental State Examination Scale (MMSE), Epworth Sleepiness Scale (ESS), and Alzheimer’s disease (AD) biomarkers before and after surgery for case group.

	Before-UPPP (*n* = 35)	After-UPPP (*n* = 27)	*P* *
Aβ42 (pg/ml)	0.23 ± 0.17	0.20 ± 0.16	0.0001
Aβ40 (pg/ml)	29.24 ± 32.52	23.52 ± 24.46	0.0046
t-tau (pg/ml)	11.88 ± 7.05	8.88 ± 6.21	0.0001
p-tau (pg/ml)	26.31 ± 14.41	20.43 ± 10.50	0.0001
Aβ42/Aβ40	0.022 ± 0.013	0.06 ± 0.018	0.032
MMSE (score)	27.14 ± 1.65	29.07 ± 1.78	0.0001
ESS (score)	11.91 ± 4.84	5.89 ± 2.83	0.0001

*P-values were calculated by using paired Wilcoxon rank-sum test.

**TABLE 3:** Differences of Alzheimer’s disease (AD) biomarkers in three groups.

	Before-UPPP (*n* = 35)	Control group (*n* = 16)	*P* *	After-UPPP (*n* = 27)	Control group (*n* = 16)	*P* *
Aβ42 (pg/ml)	0.23 ± 0.17	0.26 ± 0.22	0.631	0.20 ± 0.16	0.26 ± 0.22	0.436
Aβ40 (pg/ml)	29.24 ± 32.52	13.18 ± 10.78	0.049	23.52 ± 24.46	13.18 ± 10.78	0.291
t-tau (pg/ml)	11.88 ± 7.05	7.64 ± 4.17	0.037	8.88 ± 6.21	7.64 ± 4.17	0.651
p-tau (pg/ml)	26.31 ± 14.41	17.34 ± 9.12	0.027	20.43 ± 10.50	17.34 ± 9.12	0.466

*P-values were calculated using Wilcoxon rank-sum test.

**FIGURE 4:**
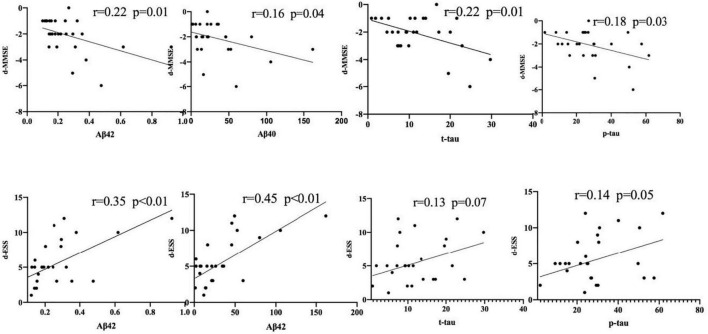
The significant negative correlations with the plasma levels of Aβ42, Aβ40, t-tau, and p-tau. The symbol ● represents the value of the patient’s biomarker.

**FIGURE 5:**
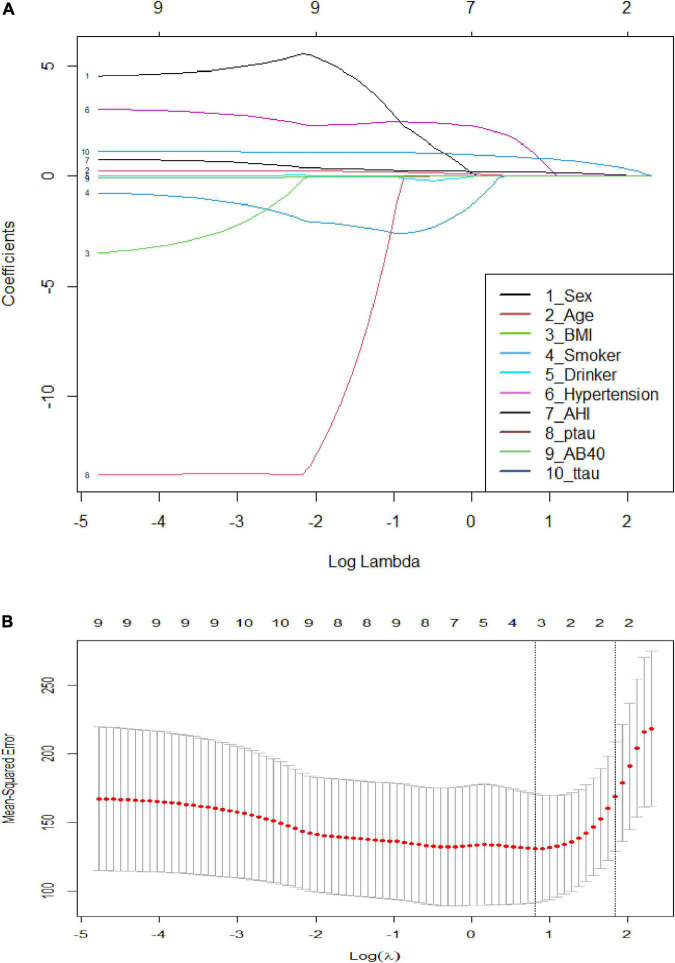
Visualized results and cross-validation of lasso regression. **(A)** Least absolute shrinkage and selection operator coefficient profiles; **(B)** Partial likelihood deviance for LASSO coefficient profiles. The red dots represent the partial likelihood values, the gray lines represent the standard error (SE), and the vertical dotted line is shown at the optimal values by 1–s.e.

A further analysis revealed that the cognitive function scores of OSAHS patients showed significant negative correlations with the plasma levels of Aβ42, Aβ40, t-tau, and p-tau, while sleepiness was significantly positively correlated with Aβ42, Aβ40, and p-tau (Figure 4). A Spearman correlation analysis revealed that the plasma levels of Aβ 42, Aβ 40, t-tau, and p-tau were significantly correlated with both cognitive function and sleepiness (*p* < 0.05) (Supplementary Figure 1). In order to study the risk factors affecting cognitive function, we constructed a linear stepwise regression model in R, the AHI, age, gender, BMI, AD biomarkers, smoking, drinking, and hypertension into the model. Gender, smoking, drinking, and hypertension were used as covariates to adjust the reliability of the model (Table 4). At the same time, we used lasso regression model to re-verify the factors that affect the outcome variable. The lasso regression results showed that AHI and t-tau protein were the influencing factors, with the significance coefficients of 0.03 and 0.31, respectively. The two model analysis results showed that AHI and t-tau were risk factors affecting cognitive function (Figure 5).

**TABLE 4:** The stepwise regression results after adding covariates.

	Estimate	Std. error	*t*-value	*P*
(Intercept)	8.375683	1.865343	4.490	0.00014
Gender	0.287285	0.337582	0.851	0.40284
Smoking	-0.100228	0.198085	-0.506	0.61730
Drinking	0.198523	0.216766	0.916	0.36850
Hypertension	-0.131112	0.191450	-0.685	0.49975
AHI	0.765769	0.083428	9.179	1.76e-09
AB42	0.014211	0.009924	1.432	0.16454
AB40	-0.004225	0.003080	-1.372	0.18236
t-tau	-3.831101	1.090859	-3.512	0.00171
p-tau	-0.023028	0.017151	-1.343	0.19145

## Discussion

Obstructive sleep apnea hypopnea syndrome is a common sleep-related respiratory disorder characterized by recurrent obstruction of the upper respiratory tract (Stuck and Maurer, 2017). Long-term chronic hypoxia induced by OSAHS may cause cognitive dysfunction and neurodegenerative diseases (Dissel et al., 2015). AD is a significant public health challenge worldwide and is associated with huge medical and economic burdens. Studies have identified hypoxia and sleep disorders as independent risk factors for AD (Zhang et al., 2007; Daulatzai, 2015; Ju et al., 2016). Therefore, AD biomarkers may accumulate in the plasma of OSAHS patients with preclinical dementia due to hypoxia and disordered sleep. Hypoxia may upregulate the shear activity of γ -secretase through the HIF-1α pathway, thus accelerating the abnormal metabolism of APP and promoting the production of Aβ (Sun et al., 2006). Hypoxia can also damage cell mitochondria and activate cell oxidative stress reactions and autophagy, and accumulated autophagosomes can continuously produce Aβ (Chen et al., 2003). Studies also have identified significant correlations between sleep disorders and AD biomarkers in patients with mild cognitive impairment or AD. Sleep disorders increase oxidative stress responses in the central nervous system and damage the structure and function of the blood brain barrier, resulting in the pathological accumulation of Aβ and tau protein. An 18-year Swedish study of 392 elderly people found that obese elderly women had a higher risk of developing dementia or AD than normal weight people. Among subjects older than 70 years, every 1.0-point increment in BMI increased the risk of developing AD by 36% (Gustafson et al., 2003). OSAHS patients often have an above-normal BMI, as in our study. Some reports mentioned that obesity may increase oxidative stress reactions and blood brain barrier damage, and described higher plasma Aβ concentrations in obese OSAHS children than in their normal weight counterparts (Zimmerman et al., 2006; Jack and Holtzman, 2013). In this study, we observed significantly higher Aβ40, t-tau, and p-tau levels in the OSAHS group relative to the control group. This difference is likely attributable to hypoxia, sleep disorders, and AHI abnormalities in OSAHS patients.

The treatment of OSAHS includes CPAP and surgery. For severe OSAHS, UPPP is the most important treatment. In previous studies, a 3-month course of CPAP significantly improved cognitive function in AD patients (Zimmerman et al., 2006; Ancoli-Israel et al., 2008). OSAHS may be a cause of cognitive functional impairment or dementia. However, the process of dementia development is reversible, and OSAHS treatment during the early stage of this process may particularly slow the progress of dementia (Ancoli-Israel et al., 2008). Similar results have been reported in studies on OSAHS children. The plasma Aβ levels in OSAHS children were significantly lower after adenotonsillectomy than before surgery, suggesting that tonsillectomy could potentially slow the progression of AD (Jack and Holtzman, 2013). Many studies of adult OSAHS have proven that CPAP therapy can reduce the risk of AD development. It remains unclear whether surgical treatment such as UPPP could also slow the progression of AD.

We observed decreases in the plasma Aβ42, Aβ40, t-tau, and p-tau levels in OSAHS patients after UPPP surgery. These changes may be related to improvements in disordered sleep and hypoxia after surgery. The MMSE evaluates mild cognitive impairment, including spatial location ability, memory ability, and calculation ability. The ESS mainly evaluates daytime sleepiness. In this study, OSAHS patients had significantly higher MMSE scores and significantly lower ESS scores after surgery relative to before surgery. These score changes were significantly correlated with the levels of Aβ42, Aβ40, t-tau, and p-tau, suggesting that these biomarkers were indeed correlated with cognitive function and sleepiness. AD mostly occurs in elderly people (>60 years of age), while the incidence of OSAHS increases with age, and even affects children and young people. Studies have shown that pathological changes associated with AD occur 15–20 years before the onset of clinical symptoms (Ganguli et al., 2014). CPAP therapy can improve the cognitive functioning of OSAHS patients. Compared with CPAP, UPPP fundamentally alleviates hypoxia and sleep disorders. In this study, the plasma Aβ42, Aβ40, t-tau, and p-tau levels decreased after UPPP. Preliminarily, we can conclude that changes in the levels of AD biomarkers can improve the cognitive functioning of patients, and that UPPP may slow the development of AD in OSAHS patients.

Studies have identified many independent factors associated with cognitive decline, including overweight, age, smoking, hypertension, and diabetes (Nugent et al., 2001; Theorell-Haglöw et al., 2006; Šimić et al., 2016; Wright et al., 2017; Sardar Sinha et al., 2018). To our knowledge, however, no previous study explored the relationships of plasma AD biomarkers (Aβ42, Aβ40, t-tau, and p-tau) with cognitive function. It has been found by PET imaging technology that during the development of AD, the signal of amyloid protein increased significantly, and OSAHS can accelerate the deposition of amyloid protein (Yun et al., 2017). The course of AD developed slowly, and the changes of relevant biomarkers occur before clinical symptoms appear (Ju et al., 2014). In OSAHS patients with normal cognitive function, the plasma levels of Aβ40, Aβ42 and total Aβ were significantly higher than those in the control group (Bu et al., 2015; Sharma et al., 2018). Even in childhood, OSAHS can accelerate the pathological process related to AD, and the level of plasma Aβ42 decreases after tonsillectomy (Kheirandish-Gozal et al., 2016). Our correlation analysis revealed that the plasma levels of Aβ42, Aβ40, t-tau, and p-tau were correlated with the MMSE and ESS scores. In the study, AHI and t-tau were potential influencing factors for cognitive decline. Aβ and Tau proteins are the key factors to promote the progress of Alzheimer’s disease, their abnormal accumulation can cause cognitive impairment (Šimić et al., 2016; Sardar Sinha et al., 2018). The effect of AHI on cognitive function is mainly due to night hypoxia. In the absence of oxygen, ADAM10 expression in nerve cells was decreased and its blocking effect on Aβ protein production was weakened (Guglielmotto et al., 2009). At the same time, hypoxia enhanced the activities of β and γ secretase by enhancing the expression of hypoxia-inducible factor 1α (HIF-1α), resulting in the increase of Aβ content in hypoxia model mice (Fisk et al., 2007; Li et al., 2009; Shiota et al., 2013). Therefore, the higher the AHI of OSAHS patients, the more obvious the night hypoxia was, and the greater the impact on cognitive function.

### This study had some limitations of note

First, the sample size was small and only included patients with severe OSAHS patients who required surgery. Therefore, we cannot verify whether the plasma levels of Aβ42, Aβ40, t-tau, and p-tau increase gradually with increasing OSAHS severity. Second, although the experimental group included 35 patients who underwent surgery, eight people were lost to follow-up. This may have introduced bias to the research results. Third, Aβ42, Aβ40, t-tau, and p-tau are more stable in cerebrospinal fluid. However, it was very difficult to collect cerebrospinal fluid from patients for this study, given the clinical risks and patient tolerance, and thus we were only able to measure the plasma AD biomarker levels. Fourthly, we did not monitor PSG of the postoperative patients and lacked sleep-related data. In a prospective intervention study, Sundman et al. (2021) followed up 65 patients with OSAHS for up to 8 years after receiving UPPP. The results showed decrease of AHI in both short-term and long-term monitoring, especially within 2 years after surgery. Boyd et al. (2013) also showed that the AHI of patients with moderate to severe OSAHS was significantly improved after UPPP. Neruntarat (2011) reported 91 patients who underwent UPPP surgery, and their average AHI decreased from 45.6 times/h at baseline to 13.4 times at the six-month follow-up, and the results were still fairly good 5 years later, with an AHI of 19.4 times/h. These studies have demonstrated the effectiveness of UPPP in reducing AHI. Unfortunately, PSG was not monitored for postoperative patients in our study. However, combined with the previous studies, it is reasonable to assume that the AD biomarkers change with the decrease of AHI. Further studies should investigate the association between the drop in AD biomarkers with decreased AHI after surgery. Fifth, we excluded patients whose parents or siblings had a history of AD. In fact, the family history of sporadic AD may not be relevant to AD-related biomarkers in patients, so we may have mistakenly excluded some patients who should be included in the study.

## Conclusion

Obstructive sleep apnea hypopnea syndrome can increase plasma AD biomarkers levels. UPPP can improve patients’ cognition and sleepiness, and the mechanism may be related to changes in plasma AD biomarkers. Higher AHI and higher t-tau level were identified as independent risk factors for cognitive decline.

## Data availability statement

The raw data supporting the conclusions of this article will be made available by the authors, without undue reservation.

## Ethics statement

The studies involving human participants were reviewed and approved by The West China Hospital Ethics Review Committees under Approval No. 2019(485). All participants gave their informed consent to participate in this study and for the use of ELISA data.

## Author contributions

WK: writing—review and editing, patient data collection included blood collection, and Elisa experiments. YZ and WK: research project design and supervision. Both authors contributed to the article and approved the submitted version.

## Footnotes

1
https://www.philips.com.cn/healthcare/

2
https://www.thermofisher.cn/cn/zh/home/about-us.html
