# Supplementary figures and images for: Alzheimer’s disease biomarkers in patients with obstructive sleep apnea hypopnea syndrome and effects of surgery: A prospective cohort study
Source: Front Aging Neurosci. 2023 Jan 17;14:959472. doi: 10.3389/fnagi.2022.959472 (PMC9887197; doi:10.3389/fnagi.2022.959472)

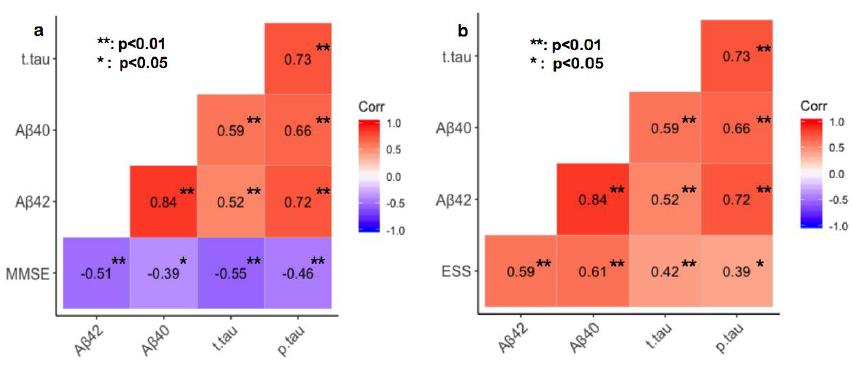

Supplement: Supplementary file 1 [file Image_1.JPEG]
